# Surgical delay in reverse sural artery flap prevents congestion of the flap: a case report of the stepwise delay method

**DOI:** 10.1080/23320885.2023.2225610

**Published:** 2023-06-20

**Authors:** Yuta Izawa, Hiroko Murakami, Tetsuya Shirakawa, Kentaro Futamura, Masayuki Hasegawa, Yoshihiko Tsuchida

**Affiliations:** a Department of Orthopedic Trauma Center, Sapporo Higashi Tokushukai Hospital, Sapporo, Hokkaido, Japan; b Department of Trauma Center, Shonan Kamakura General Hospital, Kanagawa, Japan

**Keywords:** Reverese sural artery flap, surgical delay, congestion

## Abstract

We performed reverse sural artery flap (RSAF) with the stepwise delay method, cutting the vascular pedicle step by step, as the patient had a high risk of flap necrosis. Surgical delay in RSAF is anticipated to prevent not only flap cyanosis but also flap congestion.

## Introduction

Reverse sural artery flap (RSAF) is a standard method for the reconstruction of soft tissue defects in the distal lower extremity and foot [1]. However, the originally described RSAF technique has a risk of up to 25% of partial or total flap necrosis [1,2]. Congestion is the main cause of flap necrosis, while cyanosis can also cause this disease.

Super-drainage, in which the lesser saphenous vein is anastomosed to the recipient vein, is an effective solution for congestion of the vein in RSAF [3,4]. However, microvascular anastomosis is required for super-drainage, which is not possible if no vein can be anastomosed in the recipient area. Moreover, the surgical delay has been reported to be effective in increasing the survival rate of skin flaps due to arterial network expansion [5–8] and is primarily used as a countermeasure for cyanosis of the flap. Although there is no evidence, we have occasionally observed improved congestion after surgical delay, suggesting the procedure’s potential for promoting venous drainage route development. However, flap necrosis cannot completely be avoided even with the standard surgical delay. We speculated that flap necrosis in the standard surgical delay can be attributed to the predetermined timing of vascular pedicle dissection and flap transfer, without evaluating whether sufficient arterial network expansion or venous drainage route development has been achieved. Therefore, we devised and performed a stepwise delay method, in which arteries or veins in the proximal vascular pedicle of the flap are dissected step by step while observing the color tone of the flap. We hypothesized that the stepwise delay method can be used determine the optimal timing for vascular pedicle dissection and flap transfer and would be useful for preventing flap necrosis.

In the present case, we performed RSAF with the stepwise delay method for a patient with a high risk of flap necrosis and obtained good results. In addition, we have found that surgical delay is effective not only in improving cyanosis but also in mitigating congestion.

## Materials and methods

The stepwise delay method in RSAF was performed as follows. To confirm that the RSAF can be elevated, Doppler was used to confirm the presence of the perforator of the peroneal artery, followed by ultrasonography to confirm the course of the lesser saphenous vein. The flap was designed to cover the exposed tendon and bone and to easily suture the donor site. It was elevated by keeping a 4-cm wide distal pedicle of fascia overlying the gastrocnemius muscle. The proximal vascular pedicle was exposed and secured in the proximal side of the flap. Simultaneously, the arteries, veins, and nerves in the proximal vascular pedicle were separated and identified; structures that may be included in the proximal vascular pedicle include the lesser saphenous vein, the superficial sural artery, the superficial sural vein, and the medial sural cutaneous nerve. Additionally, some cases involve only a portion of those structures. The patency of each blood vessel was confirmed, and blood vessels without patency and the medial sural cutaneous nerve can be cut at this stage. After completely elevating the flap, leaving only the proximal vascular pedicle and distal pedicle, the flap was roughly sutured to the donor site to close the wound.

Two or three days after the flap elevation, each blood vessel was clamped with a vascular  clip and the wound was closed in the operating room. Observations were made in the ward at 3, 6, and 12 h after surgery. If the flap becomes cyanotic or congested, the clip of the artery or vein was removed, respectively. If the abnormal flap color tone persists, all clips were removed, and we confirmed that the flap color tone has improved. This procedure was repeated every 2–3 d. If cyanosis did not occur even with continued clamping of the artery, it was deemed indicative of adequate blood flow development to the flap, and the artery was dissected at the next surgery. If the congestion did not occur even with continued clamping of the vein, it was deemed indicative of venous drainage development of the flap, and the vein was dissected at the next surgery. If there were multiple veins, the veins were dissected in order, starting from one with the least patency. If the color tone of the flap did not deteriorate even with the continued clamping of the last remaining artery or vein, the remaining vessels were dissected and the flap was transferred at the next surgery. The donor site was sutured and closed simultaneously. Flap elevation and flap transfer were performed under general anesthesia, while vascular clip application can be performed using a sciatic nerve block.

## Case report

**Figure 1.**
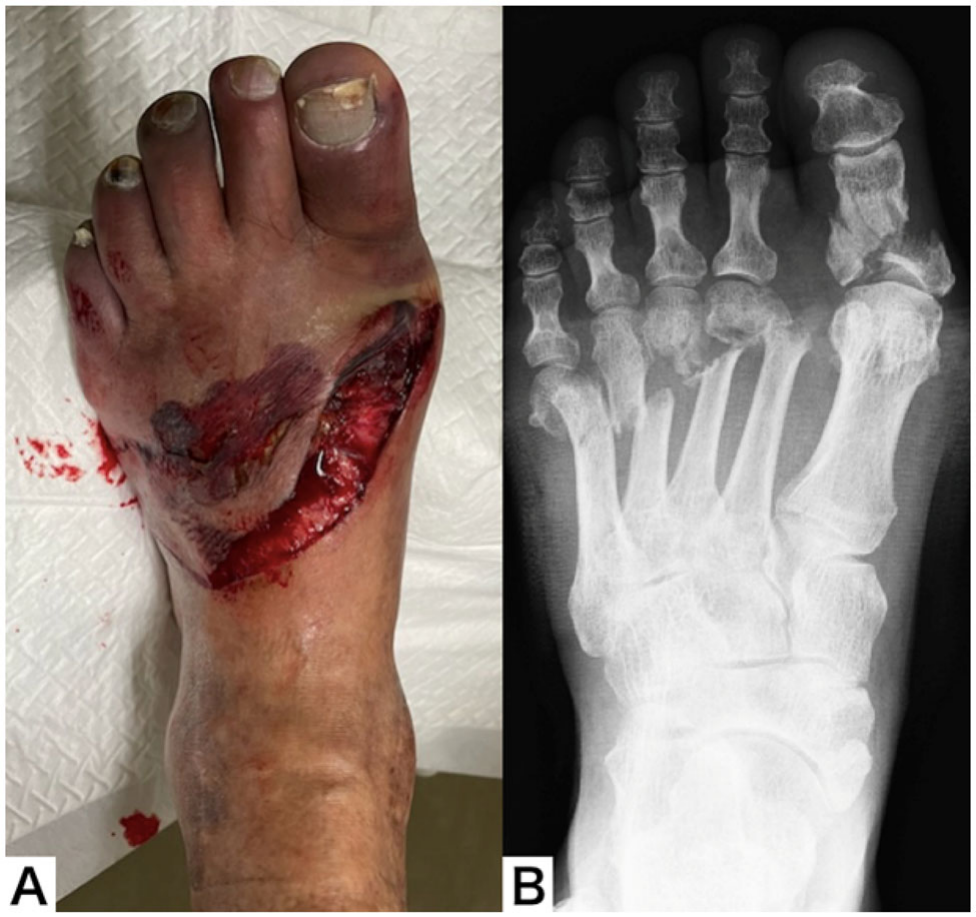
Appearance (A) and plain radiograph (B) of the left foot on Arrival at the trauma center.

A male patient aged 75 years was injured when a steel frame on his leg during factory work and was transported to our trauma center. He had a history of diabetes mellitus, chronic kidney dysfunction, arrhythmia and had a cardiac pacemaker. He was also a heavy smoker. An open wound and crushed skin were observed on the dorsum of the left foot, and the toes were purple. Ultrasound examination revealed that the dorsalis pedis artery was interrupted proximally to the open wound and the posterior tibial artery was continuous. Plain radiography revealed a proximal phalanx fracture of the big toe and 2nd to 5th metatarsal fractures (Figure 1).

**Figure 2.**
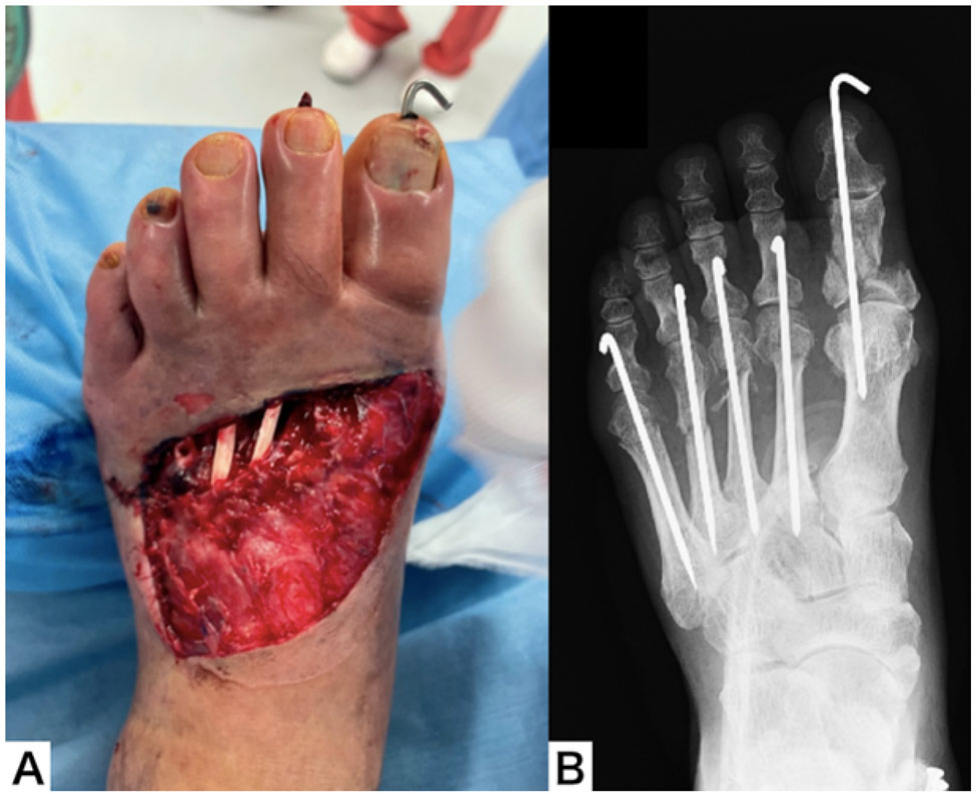
Appearance (A) and plain radiograph (B) of the left foot after Emergency operation.

Emergency surgery was performed under general anesthesia on the day of the injury. Debridement of the crushed skin on the dorsum of the foot revealed a skin defect of about 8 × 10 cm, exposing the 2nd to 5th extensor digitorum longus tendons and the metatarsal bones. After the fracture was realigned and fixed with steel wires, the poor color tone of the toes improved. Negative pressure wound therapy was performed for the skin defect (Figure 2).

**Figure 3.**
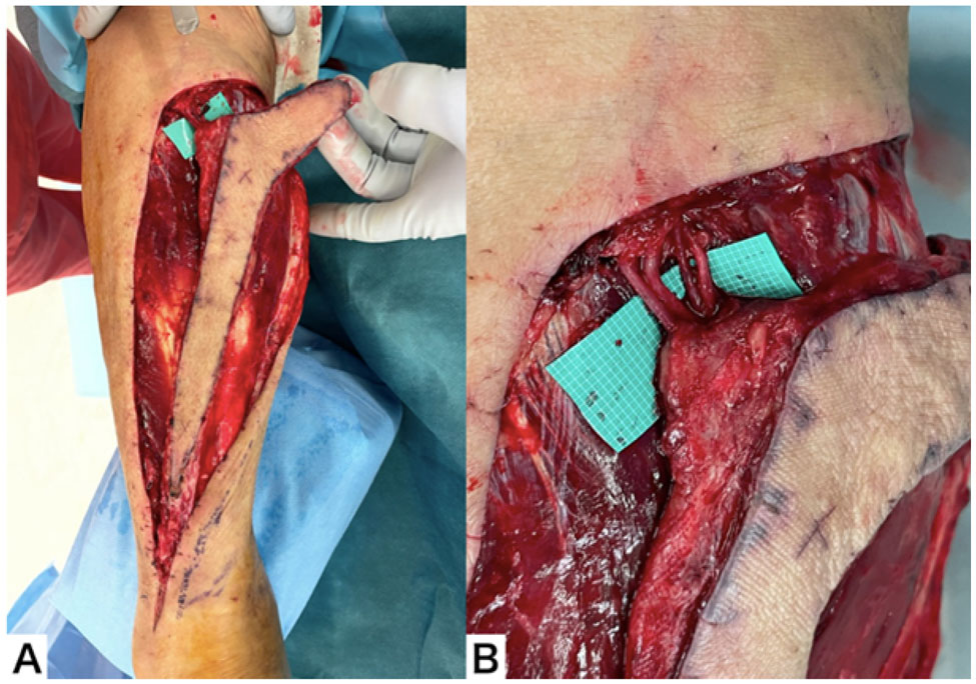
Elevation of the RSAF. (A) The flap after elevation. (B) The proximal pedicle included an artery, two veins, and a nerve.

Elevation of RASF was performed under general anesthesia two days after the injury. In order confirm that the RSAF can be elevated, Doppler was used to confirm the presence of the perforator of the peroneal artery, followed by ultrasonography to confirm the course of the lesser saphenous vein. Due to the extensive size of the skin defect area, it was judged that achieving full coverage with a flap would pose a challenge. To address this issue, a hockey-stick-shaped skin flap was designed to cover the exposed extensor tendon and metatarsal bone and to easily suture the donor site. For areas that could not be completely covered by the flap, we planned to perform skin grafting after the elevation of the granulation tissue. We unsuccessfully searched for a vein capable of super-drainage on the recipient area using ultrasound and instead decided to perform surgical delay. The proximal vascular pedicle of the flap contained the lesser saphenous vein, superficial sural artery, superficial sural vein, and medial sural cutaneous nerve, and patency was observed in each of the three vessels. The operation was terminated by rough suturing around the skin flap without any intervention on the vessels (Figure 3).

**Figure 4.**
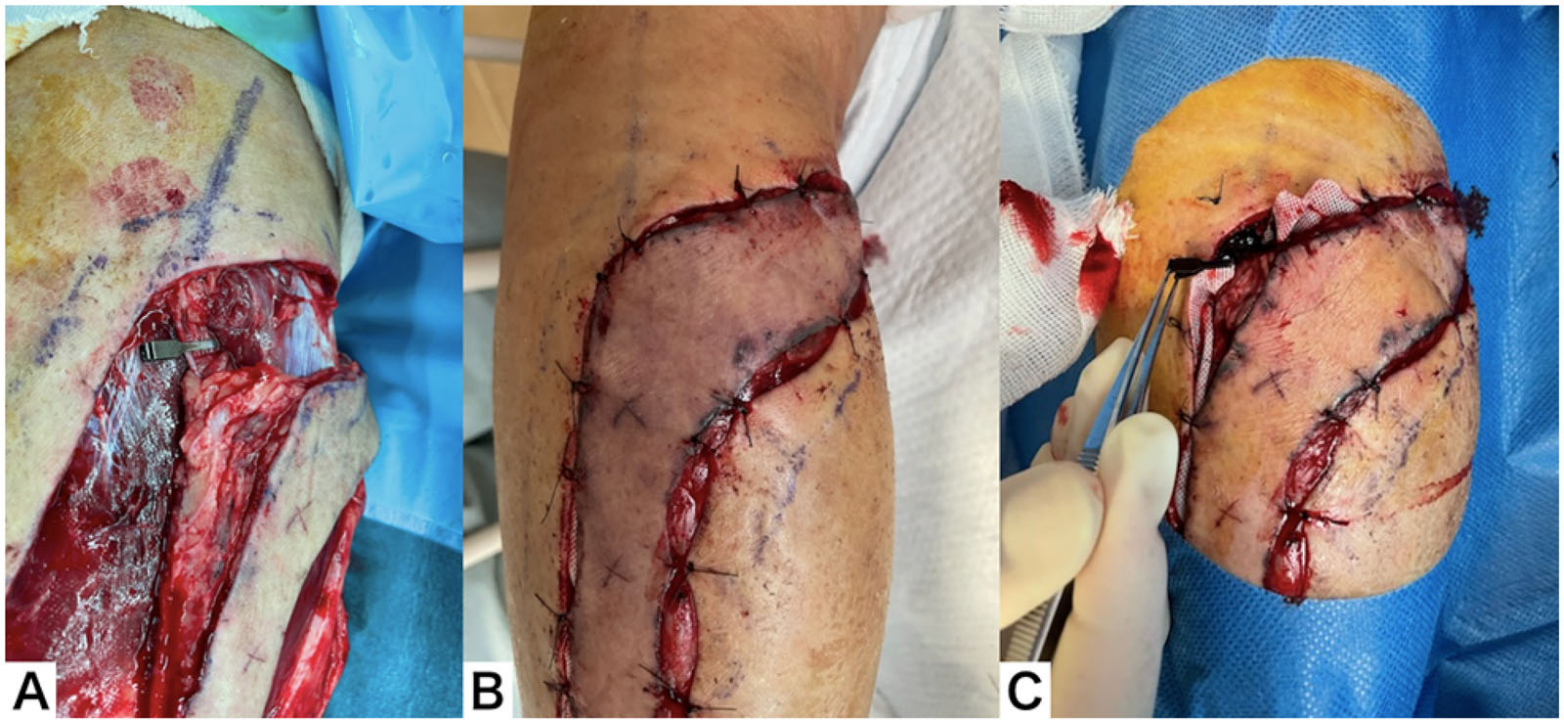
Clinical course throughout the 3 d after flap elevation. (A) The proximal pedicle was clamped with a vascular clip. (B) After 3 h, the flap was clearly congested. (C) Immediately after removing clips, the color tone of the flap normalized.

Three days after flap elevation, surgery was performed using a sciatic nerve block. The proximal vascular pedicle was clamped with vascular clips and the wound was closed. After three hours, the flap was clearly congested and had darkened margins, so the clips were removed in the ward. Immediately after removing the clips, the color tone of the flap normalized (Figure 4).

**Figure 5.**
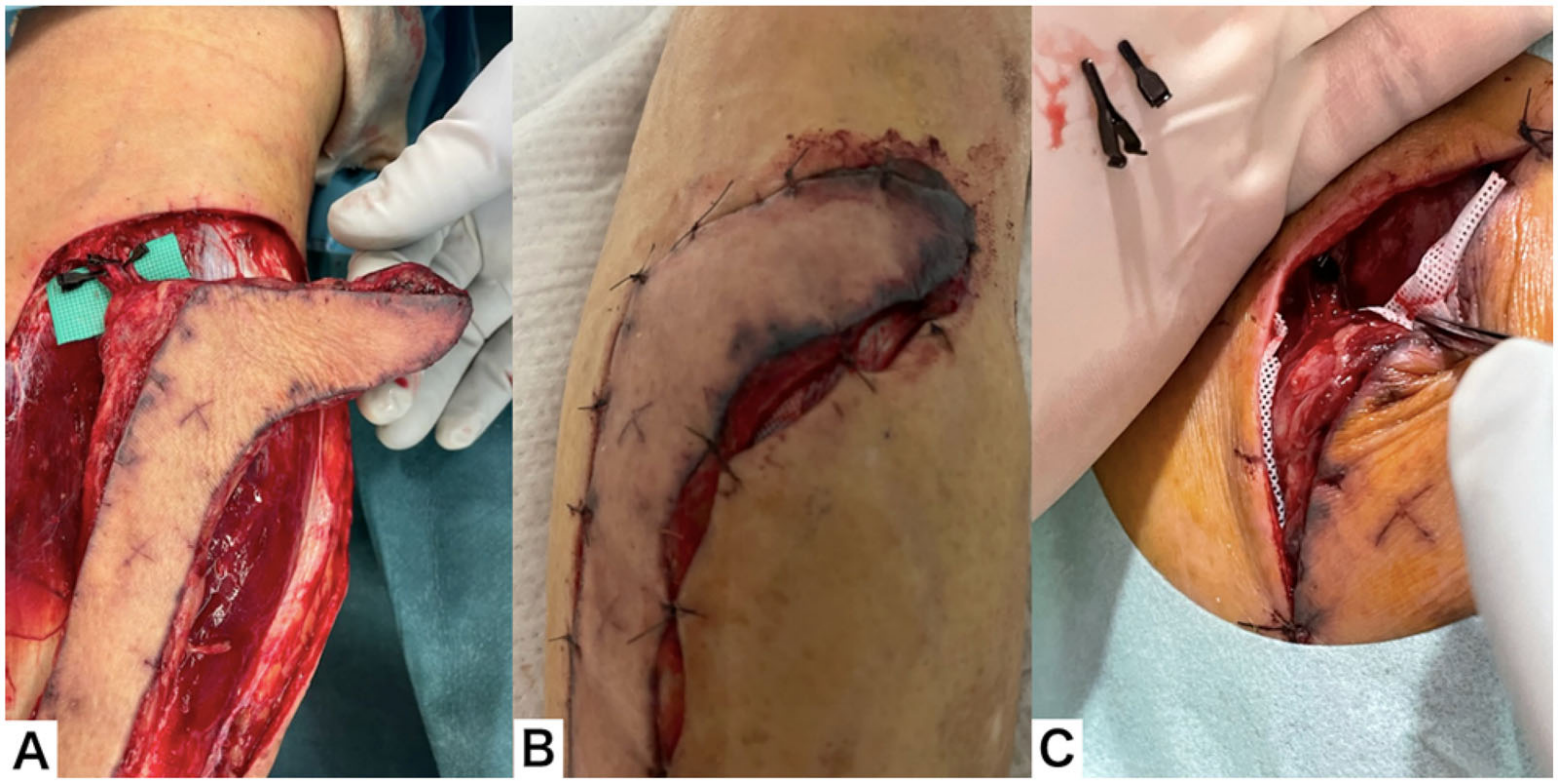
Clinical course throughout the 5 d after flap elevation. (A) Each of the three vessels in the proximal vascular pedicle was clamped with a separate vascular clip. (B) After 6 h, the flap was clearly congested. (C) Immediately after removing the clips, the color tone of the flap normalized.

Five days after flap elevation, surgery was performed using a sciatic nerve block. Each of the three vessels in the proximal vascular pedicle was clamped with a separate vascular clip, and the wound was closed. After 6 h, the flap was clearly congested. In the ward, the clips in the lesser saphenous vein and the superficial sural vein were removed, leaving only the clip in the superficial sural artery. Immediately after removing the clips, the color tone of the flap normalized (Figure 5).

**Figure 6.**
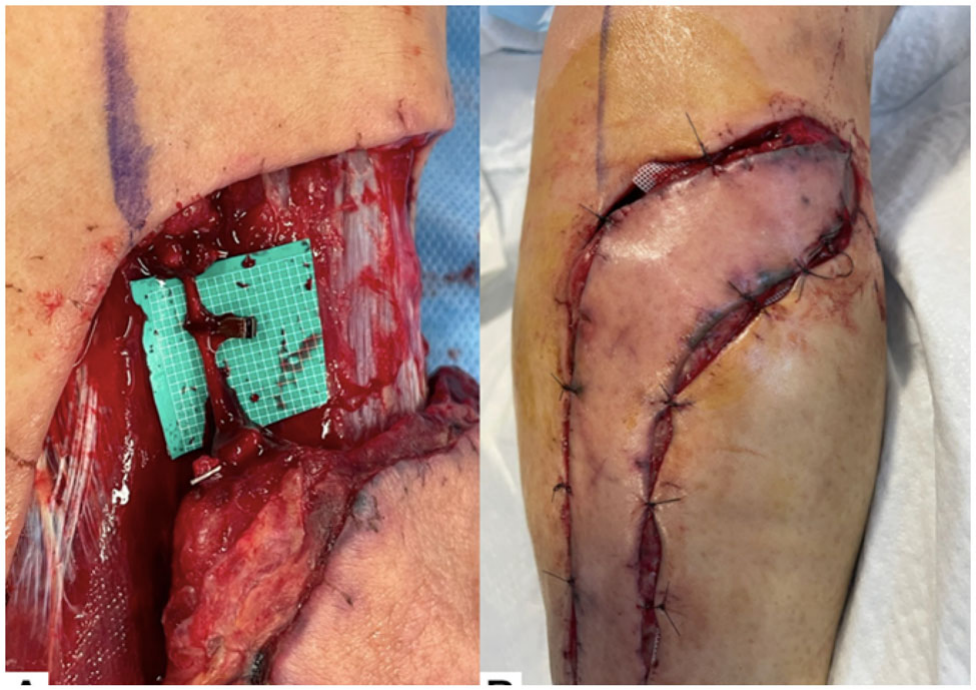
Clinical course throughout the 7 d after flap elevation. (A) The remaining superficial sural vein was clamped with a vascular clip. (B) After 6 h, the flap was clearly congested.

Seven days after flap elevation, surgery was performed using a sciatic nerve block. The patency of the superficial sural vein was good and the patency of the lesser saphenous vein was weak. The superficial sural artery, lesser saphenous vein, and medial sural cutaneous nerve were dissected. The remaining superficial sural vein was clamped with a vascular clip, and the wound was closed. After six hours, the flap was clearly congested. In the ward, the clip in the superficial sural vein was removed. Immediately after removing the clip, the color tone of the flap normalized (Figure 6).

**Figure 7.**
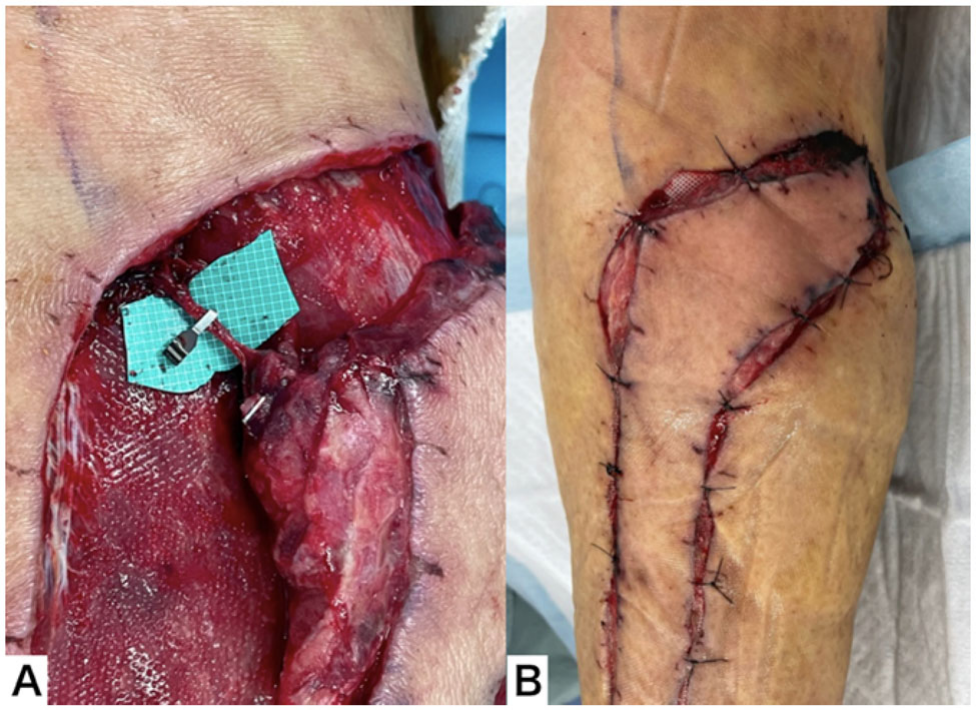
Clinical course throughout the 10 d after flap elevation. (A) The superficial sural vein was clamped with a vascular clip. (B) The flap did not become congested, even after 6 h.

Ten days after flap elevation, surgery was performed using a sciatic nerve block. The superficial sural vein was clamped with a vascular clip, and the wound was closed. The flap did not become congested even after six hours, and clamping was continued (Figure 7).

**Figure 8.**
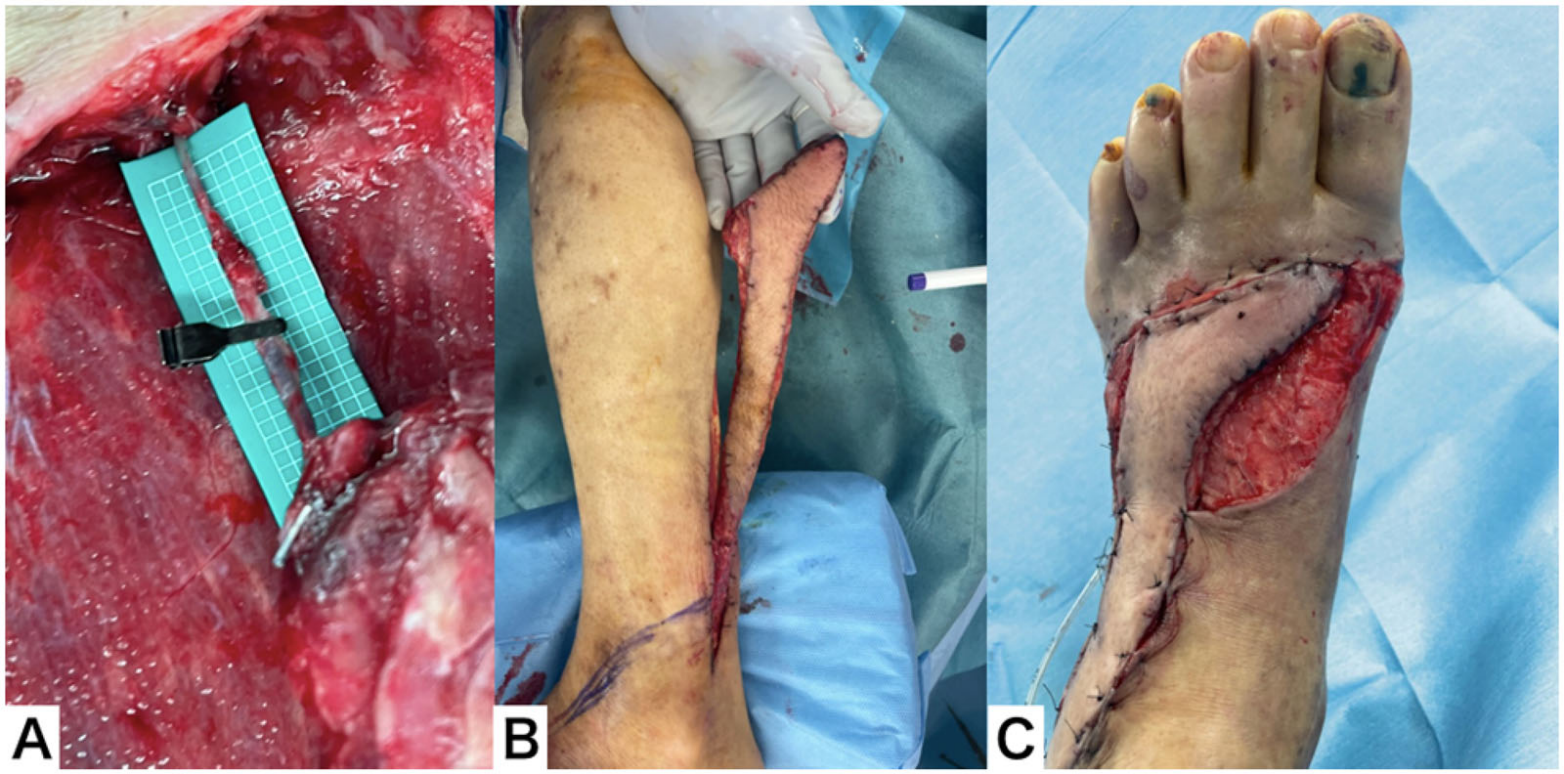
Clinical course throughout the 12 d after flap elevation. (A) The superficial sural vein was thrombosed distal to the vascular clip. (B) The superficial sural vein was dissected and the flap transferred. (C) The color of the flap remained normal after surgery.

Twelve days after flap elevation, surgery was performed under general anesthesia. The superficial sural vein was thrombosed distal to the vascular clip, but the flap color was normal. The superficial sural vein was dissected, and the flap was transferred. The donor site could be closed easily. The color of the flap remained normal after surgery (Figure 8).

**Figure 9.**
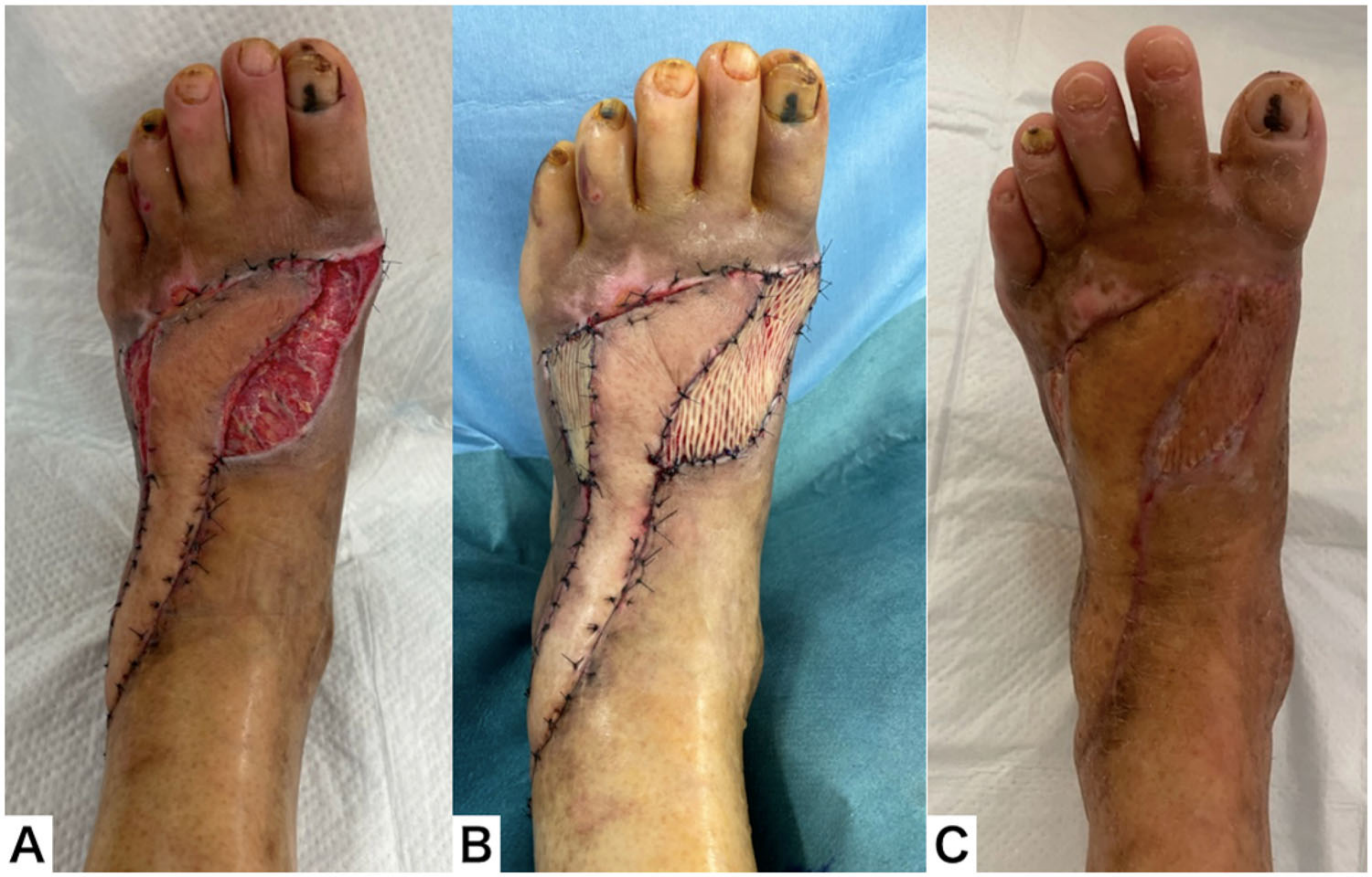
Clinical course after flap transfer. (A) The flap survived without necrosis. (B) A full thickness skin graft was performed. (C) The soft tissue healed completely 2 months after the injury.

Eighteen days after flap elevation, the flap survived without necrosis. A full-thickness skin graft was performed on the remaining raw surface area under general anesthesia. The flap survived completely, but the skin graft partially fell off. Ointment treatment was continued, and the soft tissue healed completely two months after the injury (Figure 9).

## Discussion

The patient was an elderly heavy smoker with a history of diabetes mellitus and chronic kidney dysfunction. Therefore, this patient was considered to have a high risk of partial flap necrosis associated with cyanosis and congestion. As a countermeasure against congestion, the veins on the recipient area were searched by ultrasound before surgery, but no veins that could be anastomosed were found. Therefore, we judged that RSAF using super-drainage was impossible. In addition to the lesser saphenous vein, this patient also had a relatively thick superficial sural artery and superficial sural vein, and patency was confirmed in each vessel. Therefore, we performed the stepwise delay method, in which the proximal vascular pedicle was cut step by step to manage both flap cyanosis and congestion. In our method, the vessels were temporarily clamped with vascular clips in the operating room, and the clips were removed in the ward when the color of the flap deteriorated. When the deterioration in the color tone of the flap, resulting from the clamping of blood vessels, ceases to occur, it can be judged that a sufficient delay effect has been obtained. Many methods have been reported to improve the flap survival rate in RSAF, including modification of flap design, use of subcutaneous tunnels, super drainage, and surgical delay. However, the stepwise delay method is the only method that allows prior to flap transfer the evaluation of whether flap cyanosis and congestion can occur. Despite its drawbacks, including the additional burden it places on both patients and surgeons due to multiple surgeries and meticulous flap monitoring, it is a valuable approach, particularly for patients with a high risk of flap necrosis. A prospective study including a large number of cases is required to demonstrate the clinical efficacy of the stepwise delay method.

The main purpose of the surgical delay is to expand the arterial network as a countermeasure against cyanosis. In our case, the flap did not show cyanosis and became congested due to the clamping of the vascular pedicle. With the passage of time from flap elevation, the time from vascular clamping to congestion was prolonged, and the congestion eventually disappeared. Therefore, the venous drainage route likely developed as a result of the surgical delay. The repeated clamping of the vessels until unacceptable congestion occurred also contributed to the development and training of the venous drainage route. Venous drainage in RSAF is thought to be due to an avalvular concomitant venous network [9–12]. In this case, when the flap was elevated, the superficial sural vein and the lesser saphenous vein made up the main venous drainage route. Therefore, we can infer that a venous drainage route that does not go through the superficial sural vein and the lesser saphenous vein is established by strengthening the avalvular concomitant venous network by stepwise delay. The course of our case suggests that surgical delay helps prevent congestion in RSAF. However, to confirm our findings, further evidence from animal studies is needed.

We performed RSAF with the stepwise delay method, cutting the vascular pedicle step by step, as the patient had a high risk of flap necrosis. Surgical delay in RSAF is expected to prevent flap congestion. In addition, the stepwise delay is useful for preventing flap necrosis, as it enables surgeons to determine the safe timing for vascular dissection and flap transfer.
